# Decortication with uniport video-assisted thoracoscopic surgery for empyema due to postoperative esophageal leakage: a report of two pediatric cases

**DOI:** 10.1186/s40792-024-02049-8

**Published:** 2024-10-28

**Authors:** Yudai Goto, Seiya Ogata, Hirofumi Shimizu, Michitoshi Yamashita, Takuya Inoue, Takeo Hasegawa, Yutaka Shio, Hiroyuki Suzuki, Hideaki Tanaka

**Affiliations:** 1 Department of Pediatric Surgery, Fukushima Medical University Hospital, 1 Hikarigaoka, Fukushima, Fukushima 960-1295 Japan; 2 Department of Chest Surgery, Fukushima Medical University, Fukushima, Japan; 3 Department of Pediatric Surgery, Faculty of Medicine, University of Tsukuba, Tsukuba, Japan

**Keywords:** Decortication, Empyema, Child, Video-assisted thoracoscopic surgery, Esophageal leakage

## Abstract

**Background:**

Video-assisted thoracoscopic surgery (VATS) is considered useful for the treatment of parapneumonic empyema in children. However, thoracoscopic management of empyema due to esophageal leakage as an operative complication has not been well described in the literature.

**Case presentation:**

We successfully decorticated severe empyema using uniport VATS in 2 children (a 2-year-old boy who suffered esophageal perforation after laparoscopic anti-reflux surgery, and a 7-month-old girl who had anastomotic leakage after thoracoscopic repair of esophageal atresia). In these patients, we noticed that pleural effusion rapidly progressed to empyema and caused respiratory insufficiency due to wide-range coverage by fibrotic pleural rind that was successfully decorticated under video-assisted vision from a mini-thoracotomy, followed by spontaneous healing of the leakage.

**Conclusions:**

We did not attempt to closely approach or try to repair the esophageal leakage. We believe that this is an important tip for these situations.

**Supplementary Information:**

The online version contains supplementary material available at 10.1186/s40792-024-02049-8.

## Introduction

Video-assisted thoracoscopic surgery (VATS) for empyema in children has been considered useful, mainly for the treatment of parapneumonic empyema [1]. However, thoracoscopic management of complicated empyema due to esophageal leakage as an operative complication has not been well described in the literature [2]. We herein report two pediatric cases of empyema after anti-reflux surgery and thoracoscopic repair of esophageal atresia that were treated successfully with decortication using uniport VATS, with satisfactory outcomes achieved.

## Case presentation

### Case 1

**Fig. 1:**
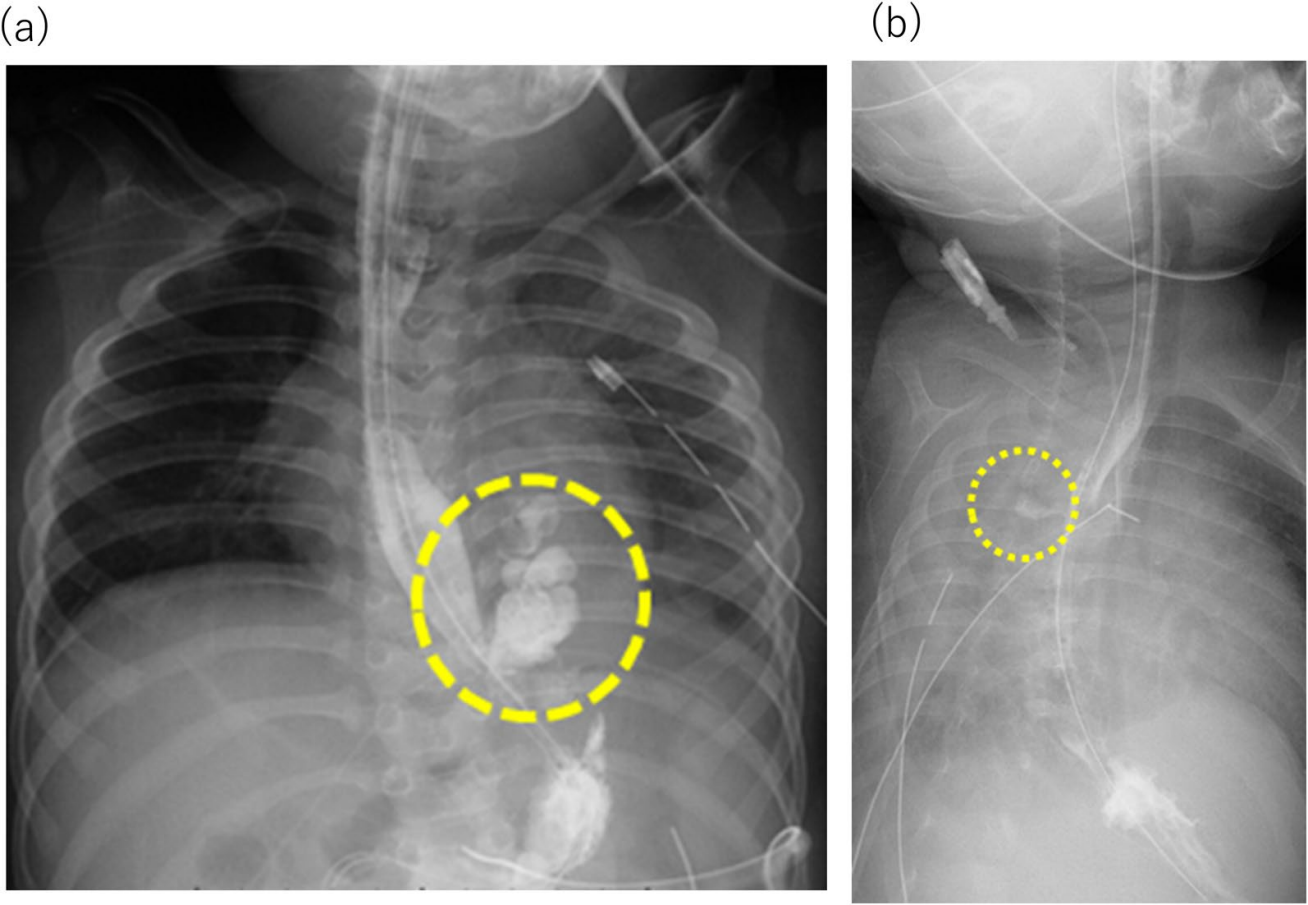
Esphagograms in Case 1 (**a**) and 2 (**b**) show leakage from the lower esophagus and the anastomosis, respectively (dotted circle)

**Fig. 2:**
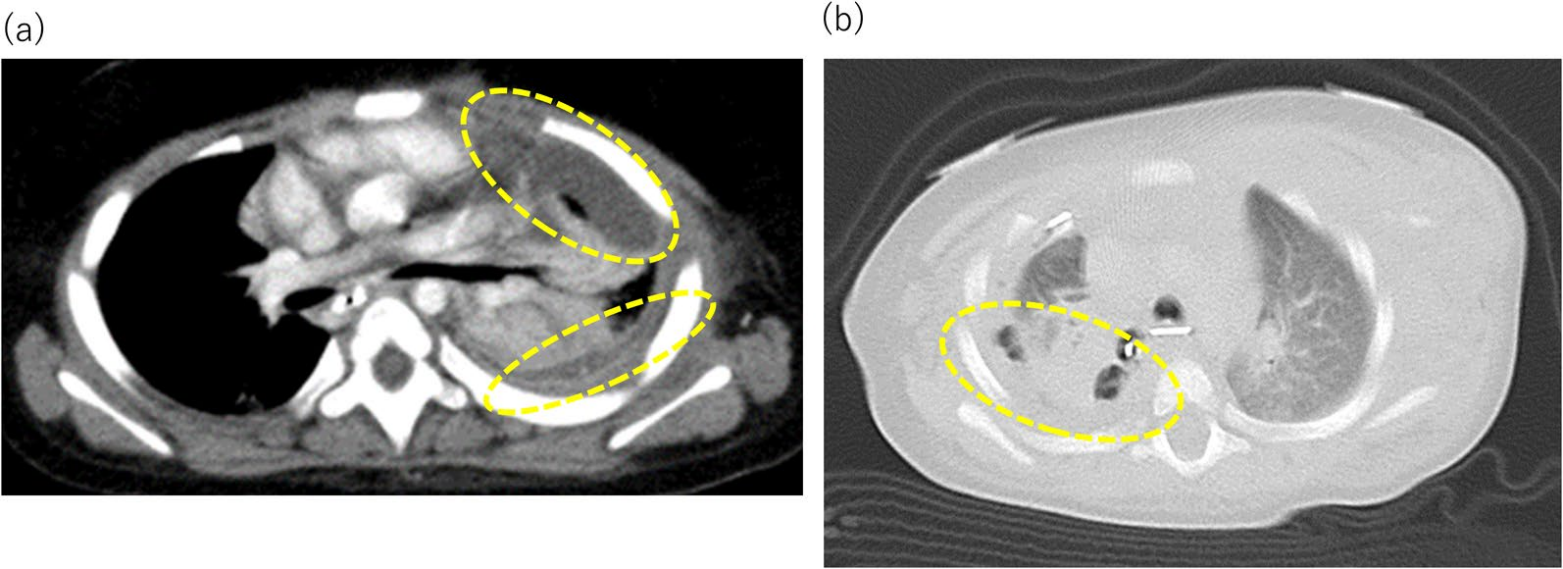
Preoperative computed tomography scans of the chest in Cases 1 (**a**) and 2 (**b**) show a large amount of air-containing fluid collection with loculation in the left and right pleural space (dotted circle), respectively

**Fig. 3:**
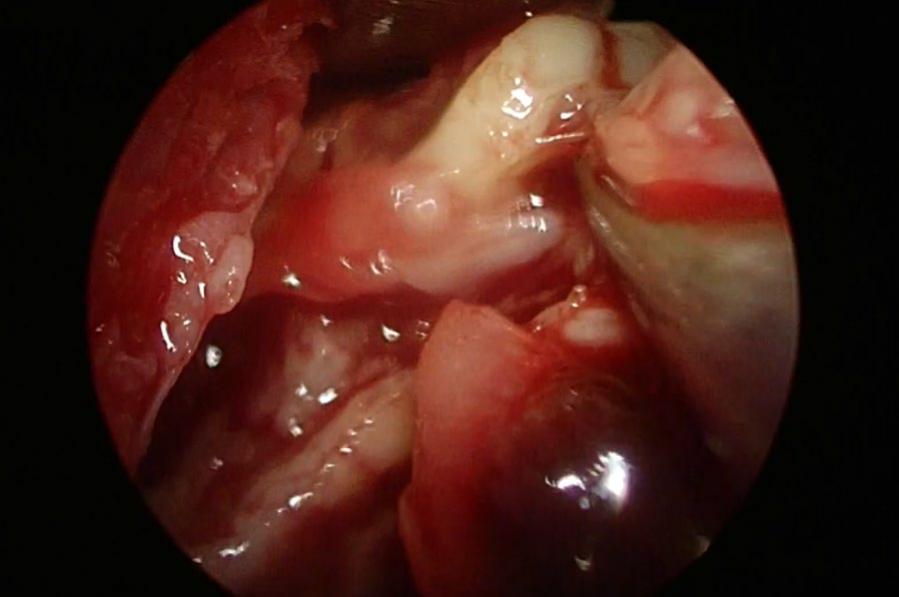
Fibrous rind was removed with curved forceps under video assistance in Case 1

A 2-year-old boy with gastroesophageal reflux underwent laparoscopic fundoplication, during which the left lateral wall of the esophagus was slightly torn when sutured to the left crus of the diaphragm and repaired. A subphrenic drain was placed before the end of the surgery. Chest radiography revealed a large amount of left pleural effusion on postoperative day (POD) 2, and esophagography revealed perforation of the left wall of the esophagus with contrast medium leaking into the left pleural cavity (Fig. 1a). Although a chest tube (12 Fr) was promptly placed in the left pleural space from the fifth intercostal space at the midaxillary line, he continued to have atelectasis on chest radiography and required ventilator support. Computed tomography (CT) revealed a large amount of air-containing fluid with loculation in the left pleural space, indicating empyema (Fig. 2a). The bacterial culture results from the drainage fluid showed Bacteroides vulgatus and Eikenella corrodens. Despite treatment with antibiotics, the patient remained febrile, and the empyema did not improve despite placement of another chest tube (12 Fr) from the sixth intercostal space. As the esophageal leakage was still considered active, decortication was performed on POD 9 (Video 1). VATS was performed with the patient in the right-lateral decubitus position. We attempted to place the first port through one of the drain sites, but we could not do so because of dense adhesions throughout the affected intrathoracic cavity. A mini-thoracotomy of approximately 4 cm was then created in the 6th intercostal space, and blunt dissection was performed using the operator’s finger. Video-assisted dissection was started using Cherry Dissectors (Ethicon Endo-Surgery, Co., Ltd., Cincinnati, OH, USA), long curved forceps, and electrocautery inserted through the incision (uniport VATS). While we broke down the loculations and debrided the fibrinous pyogenic materials (Fig. 3), the lung was covered by a fibrous rind, which was mostly removed with video assistance, regaining satisfactory expansion of the left lung. We did not attempt to closely approach or repair esophageal leakage. A small injury to the surface of the lung was sutured and the operation was terminated with two chest tubes. Irrigation of the left pleural cavity was continued postoperatively, and the esophageal perforation was confirmed to have healed with esophagography. He was extubated 10 days after VATS and discharged 40 days after VATS.

### Case 2

A 7-month-old girl underwent thoracoscopic repair for long-gap esophageal atresia (Gross type A) after elongation of both the upper and lower ends of the esophagus (Foker’s method). Anastomotic leakage was confirmed by esophagography on postoperative day POD 7 (Fig. 1b). The original 10-Fr chest tube inserted from the fourth intercostal space at the time of esophageal anastomosis did not seem to be effective and was replaced by a 12-Fr tube. The addition of another chest tube (10 Fr) from the sixth intercostal space to drain the right pleural effusion and serial irrigation of the pleural cavity resulted in a limited success. The bacterial culture of the drainage fluid revealed *Corynebacterium* spp. Despite the effectiveness of antibiotic therapy, CT revealed air-containing loculated fluid collections on the dorsal side of the right pleural space, indicating empyema (Fig. 2b), and esophageal leakage seemed persistent. Decortication was considered necessary, and uniport VATS was performed on POD 14 with a 5-cm mini-thoracotomy. Breaking down the loculations, debridement of fibrinous pyogenic materials, and removal of the fibrous rind covering the right lung led to satisfactory expansion of the right lung. Similar to Case 1, we did not attempt to closely approach or repair the esophageal leakage and ended the procedure with the placement of two chest tubes. Esophageal leakage ceased thereafter, and the patient was extubated 16 days after VATS, followed by an uneventful postoperative recovery.

## Discussion

To our knowledge, this is the first report that describes the technical details of uniport VATS for the decortication of empyema that occurred after postoperative esophageal leakage in pediatric patients. Both medical and surgical treatments play significant roles in the management of parapneumonic effusion (pleural effusion associated with pneumonia) in children [3]. The usefulness of VATS compared with medical treatments, including intrapleural fibrinolytics, for the treatment of parapneumonic empyema in children has been proven in many studies in that thoracoscopic visualization of the pleural cavity permits efficient debridement, thorough adhesiolysis, and optimal placement of drainage tubes [4], as well as minimal invasion, less postoperative pain, faster recovery, and avoidance of long-term sequelae of thoracotomy, such as chest wall deformity [5]. However, these discussions have never been pursued from the viewpoint of empyema as a complication of esophagus-related surgery.

Whether open or thoracoscopic techniques are used, anastomotic leakage at the esophagoesophagostomy site occurs in approximately 13–16% of patients with congenital esophageal atresia [6]. Major disruptions rarely occur, leading to mediastinitis and/or empyema, when uncontrolled by drainage using chest tubes and antibiotics. The strategy for empyema in this setting using VATS has not been well described in the literature, although there have been a few case series studies describing the early repair of anastomotic leakage with VATS after corrective surgery for esophageal atresia [7] and mediastinal drainage for iatrogenic esophageal perforations [8]. Fundoplication, on the other hand, has been widely performed in children with refractory gastroesophageal reflux, and esophageal perforation noted during or after the operation has rarely been described in the relevant literature [9, 10]; however, leakage to the pleural cavity has never been reported. Adequate drainage of pyogenic fluid and contaminated digestive juice as well as expansion of the lung with chest tubes would allow esophageal leakage to be spontaneously cured in these situations, while inadequate drainage even with additional chest tubes would likely lead to the rapid development of empyema, which we believe occurred in our presented cases. Larger-diameter tubes might have worked more effectively in our cases, although the size of the chest tubes was limited, depending on the width of the intercostal spaces in small infants. Compared to parapneumonic empyema, which starts from an uncomplicated effusion that is often initially sterile and gradually progresses to the fibropurulent stage [1], esophageal leakage-related empyema may develop much faster, as it involves bacterial contamination from the initial presentation.

Our patients underwent decortication on POD 9 and 7, respectively, and we did not attempt to repair the leakage because exposure of the leaking site with a high degree of tissue inflammation seemed difficult and hazardous. If we had approached the esophageal leakage thoracoscopically much earlier, such as with the time lapse between occurrence of the leakage and the intervention of less than 24 h, primary repair of the leaking site might have been an optimal procedure, similar to the setting of Boerhaave’s syndrome [11]. Furthermore, if we had performed reoperation a few days earlier thoracoscopically, such as on POD 3 or 4, we could not have repair the leakage primarily; however, the procedures would have been simpler and less invasive with adequate irrigation of the leakage site and placement of large-bore chest tubes in suitable positions.

Manipulations during VATS in pediatric patients are generally more difficult than in adults in that children have more delicate and smaller anatomies and more limited working space for the operator, making each procedure technically demanding with smaller instruments necessary. Obtaining single-lung ventilation is necessary for appropriate visualization of meticulous procedures, especially in the setting of lung lobectomy for congenital cystic lung diseases in small children, thus requiring pediatric anesthetic expertise. In addition to these situations in children, uniport VATS through a small incision with meticulous procedures would be feasible because of the wide area of dense adhesions in empyema.

## Conclusions

There are two important points when performing VATS for empyema after esophageal leakage. First, the operator should not attempt to perform the procedure completely thoracoscopically because insertion of the first trocar is difficult due to dense adhesion under the rib cage. Rather, a small thoracotomy should be performed first, from which blunt dissection can be started using a finger and/or a Cherry Dissector to safely ensure sufficient space for the insertion of the next port if needed. Second, esophageal leakage should not necessarily be sutured to stop the leakage because it may worsen the situation; rather, careful and appropriate dissection and debridement with placement of a new drainage tube close to the leakage point is sufficient and can lead to spontaneous healing of the leakage, as was confirmed in the present cases.

## Supplementary Information

Supplementary Material 1: Video 1. Edited operative video of decortication using uniport VATS in Case 1. Video-assisted blunt dissection and pyogenic material were debrided using the operator’s finger and forceps to regain satisfactory expansion of the left lung. A small injury to the surface of the lung was sutured and the surgery was terminated with chest tubes.

## Data Availability

Data sharing is not applicable to this article as no datasets were generated or analyzed during the current study.

## Abbreviations

VATS: Video-assisted thoracoscopic surgery
POD: Postoperative day
CT: Computed tomography
